# Proteomic Assessment of Biochemical Pathways That Are Critical to Nickel-Induced Toxicity Responses in Human Epithelial Cells

**DOI:** 10.1371/journal.pone.0162522

**Published:** 2016-09-14

**Authors:** Yue Ge, Maribel Bruno, Najwa Haykal-Coates, Kathleen Wallace, Debora Andrews, Adam Swank, Witold Winnik, Jeffrey A. Ross

**Affiliations:** National Health and Environmental Effects Research Laboratory, US Environmental Protection Agency, Durham, NC, 27711, United States of America; Shantou University Medical College, CHINA

## Abstract

Understanding the mechanisms underlying toxicity initiated by nickel, a ubiquitous environmental contaminant and known human carcinogen is necessary for proper assessment of its risks to human and environment. Among a variety of toxic mechanisms, disruption of protein responses and protein response-based biochemical pathways represents a key mechanism through which nickel induces cytotoxicity and carcinogenesis. To identify protein responses and biochemical pathways that are critical to nickel-induced toxicity responses, we measured cytotoxicity and changes in expression and phosphorylation status of 14 critical biochemical pathway regulators in human BEAS-2B cells exposed to four concentrations of nickel using an integrated proteomic approach. A subset of the pathway regulators, including interleukin-6, and JNK, were found to be linearly correlated with cell viability, and may function as molecular determinants of cytotoxic responses of BEAS-2B cells to nickel exposures. In addition, 128 differentially expressed proteins were identified by two dimensional electrophoresis (2-DE) and mass spectrometry. Principal component analysis, hierarchical cluster analyses, and ingenuity signaling pathway analysis (IPA) identified putative nickel toxicity pathways. Some of the proteins and pathways identified have not previously been linked to nickel toxicity. Based on the consistent results obtained from both ELISA and 2-DE proteomic analysis, we propose a core signaling pathway regulating cytotoxic responses of human BEAS-2B cells to nickel exposures, which integrates a small set of proteins involved in glycolysis and gluconeogenesis pathways, apoptosis, protein degradation, and stress responses including inflammation and oxidative stress.

## Introduction

Nickel compounds are present in various industrial and environmental exposures [1, 2]. They are ubiquitous, highly toxic, and carcinogenic, and pose serious environmental and human health concerns. Humans are exposed to nickel compounds mainly by inhalation, and nickel inhalation results in a variety of adverse health effects, particularly of the respiratory system, such as acute lung injury caused by respiratory epithelial cell damage and loss of function [3]. The initial lung injury triggers the production of growth factors, extracellular matrices, and cytokines, and stimulates inflammatory responses [4]. The type of protein responses and extent of signaling pathway activation are critical to, or even determine the magnitude of nickel-induced toxicity and severity of acute lung injury. The carcinogenicity of nickel is well documented [3–5]. Many types of malignant tumors are associated with nickel exposures. Skin and lung cancer are probably more susceptible to nickel exposures as compared to other nickel-induced cancers since epithelial cells are usually the targeted sites of nickel exposures [3–5]. However, molecular mechanisms and pathways that are critical to nickel-induced toxicity and diseases are still uncertain.

Previous studies showed that nickel compounds are not strong mutagen [3–5]. It has been reported that nickel exerts its toxic and carcinogenic activity via induction of oxidative stress, generation of genomic instability by interfering with DNA repair systems, dysregulation of oncogenes or tumor suppressor proteins, and disruption of signaling pathways including modulation of enzymes and transcription factors [6–9]. Many proteins involved in cell growth, apoptosis, oxidative stress response, and inflammation, including hypoxia inducible factor 1 alpha (HIF-1α), p53, and NFκB are differentially modulated by exposures to nickel [6, 1, 2, 9]. In the nickel-mediated hypoxia signaling pathway, HIF-1α is stabilized, and the accumulated HIF-1α subsequently modulates the expression of downstream genes involved in proliferation, survival, tumorigenesis, glucose transport, and glycolysis. [6, 10]. Cells exposed to nickel maintain a high glycolytic rate and thereby acquire a metabolic phenotype similar to that of cancer cells [1]. The tumor suppressor p53 controls a number of key events leading to either DNA repair processing, cell cycle arrest, or apoptosis via coordinated pathways [11], and dysregulation of p53 is linked to many human cancers including nickel-induced carcinogenic processes [11–12]. Mechanisms of p53 activation in response to nickel and other carcinogenic metals include oxidative stress, controls of cytokine production, cell growth and differentiation, angiogenesis, and impaired p53-DNA binding capacity [12]. Nickel also affects cell growth by mechanisms such as changes in expression of growth-related factors and inactivation of growth regulation [1, 2, 13]. In addition, nickel compounds modulate several signal transduction pathways, such as the mitogen-activated protein kinase (MAPK), Akt, and glycogen synthase kinase 3β (GSK3β) pathways [12, 14]

Given the available evidence, it has been clear that the health status of nickel-exposed human cells is largely determined by the expression, posttranslational modification, and activity levels of multiple proteins that provide the molecular bases for cellular toxicity and phenotypic changes resulting from the exposures. Thus, proteome-wide assessment of these protein responses and pathways and linkage to phenotypic changes is needed to fully characterize the mechanisms by which nickel exerts its toxic effects. In the present study, we measured expression and phosphorylation status of 14 key regulators involved in metal toxicity pathways using multiplexed ELISA, two dimensional gel electrophoresis (2-DE), and mass spectrometry (MS) in human airway epithelial cells, BEAS-2B cells exposed to four concentrations of nickel chloride (Ni II) in order to identify critical protein or pathway determinants of cytotoxic responses of BEAS-2B cells to Ni (II) treatment.

## Materials and Methods

BEAS-2B cells were purchased from American Type Culture Collection (Rockville MD, USA). Trypsin Gold (MS-grade) was obtained from Promega (Madison, WI, USA). Dithiothreitol (DTT) was a product of Bioanalytical (Natick, MA, USA). Non-linear immobilized pH gradient (IPG) strips (pH 3–11), CyDyes (Cy3, Cy2 and Cy5), Typhoon 9410 scanner, Ettan IPGPhor^™^ apparatus, Decyder software, and 2D Image Quant (version 5.1) were obtained from GE Healthcare (Piscataway, NJ, USA). Methanol (HPLC grade) and glacial acetic acid were purchased from Fisher Scientific (Fair Lawn, NJ, USA). Protean II apparatus was a product from Bio-Rad (Hercules, CA, USA). Protease/phosphatase inhibitors were purchased from Calbiochem (San Diego, CA, USA). IPA Software was purchased from Ingenuity Systems (Redwood City, CA, USA). LHC-9 medium, HEPES buffer, saline solution (HBSS) and trypsin neutralizing solution (TNS) were obtained from Lonza (Walkersville, MD, USA). Versene, TrypLE^™^, Dulbecco’s phosphate buffer solution (DPBS) and Sypro Ruby stain were obtained from Life Technologies (Carlsbad, CA, USA). Precast 8–16% gradient polyacrylamide gels were from Jule, Inc (Milford, CT, USA). The Akt, Human Proinflammatory-9 Plex, HIF-1α, Apoptosis, MAPK and EGFR ELISA kits for measurement of protein changes at expression and phosphorylation levels in the treated BEAS-2B cells were from Meso Scale Discovery, Gaithersburg, MD, USA). Hoechst and Alexa-fluor 488 dyes for apoptosis staining were from Thermo Scientific (Fremont, CA, USA). The human metallothionein 2 kit (Cat. No. MBS703385) was from MyBiosource (San Diego, CA, USA). Ni (II), trypan blue, neutral red and all other chemicals were obtained from Sigma-Aldrich (St. Louis, MO, USA). Ni (II) was solubilized in deionized water for cell treatments.

### Treatment of BEAS-2 B cells with Ni (II) and cytotoxicity assays

BEAS-2B human bronchial epithelial cells (American Type Culture Collection, Rockville, MD) were cultured in LHC-9 medium as previously described [15]. Cells were treated with 30, 60, 75, or 100 μM of Ni (II) and incubated for 48 hr at 37°C and 5% CO_2_ before harvest from 96-well plate for measurements of cytotoxicity using a neutral red assay [15] and from 150 mm flasks for analysis of protein expression and phosphorylation changes using multiplexed ELISA and 2-DE analysis of protein expression and phosphorylation changes. For cytotoxicity assay, 96-well plates were seeded at a density of 2.5×10^4^ cells/well (in 0.25ml medium/well). Each plate had a blank and negative control column. After 48 hr of growth, cells were dosed with Ni (II), which was serially diluted from the high-dose concentration in subsequent microplate columns for 48 hr. After the incubation period, the wells were washed with DPBS. Neutral Red media [LHC-9 with 0.003% Neutral Red] was added, and cells were then incubated for 3 hr. Neutral Red media were removed, and wells were again washed with DPBS before the extraction buffer (50% ethanol and 1% acetic acid) were added to each well. Plates were shaken for 20 min and read at 540 nm to measure dye uptake. The measured Neutral Red dye uptake was used to calculate the percentage of survival where percentage of survival = (A540 treatment/A540 control) ×100. All experiments were triplicated with six independent cell passages. Relative cell survival was calculated as the ratio of living cells after exposure to Ni (II) divided by the number of living cells in concurrent untreated controls (p <0.05).

### Apoptosis staining and visualization

After the 48 hr incubation with NI (II) at the four different concentrations, BEAS-2B cells were fixed and nuclei stained with 4% paraformaldehyde/ Hoechst 33342 dye solution, and permeabilized with 200 μL of 1X PBS/0.1% Triton-X100 for 30 min at room temperature in 96-well plate according to manufacturer’s instructions. After this, the wells containing the fixed cells were washed with binding buffer (1XPBS/ 1% BSA) and then aspirated. F-actin in cells was then stained with 50 μL of Alexa^®^ Fluor 488 Phalloidin after a 20 min incubation at room temperature, and were then rinsed with 100 μL of 1×PBS.

### Isolation of total proteins from BEAS-2B cells for 2-DE gel electrophoresis

After the removal of the control and treated BEAS-2B cells from the incubator, 10 ml of ice-cold washing buffer (250 mM sucrose/10 mM Tris solution, pH 7.4) were added to the cell cultured dishes. Cells were then scraped off from the flasks, transferred to 15 ml conical tubes, and centrifuged at 1300 x g for 5 minutes at 4°C. Cells were washed three times by suspending in ice-cold washing buffer, centrifuging at 1300 × g for 10 min, and then lysed in 0.5 ml of 2-DE sample buffer containing 30 mM Tris (pH 8.8), 2M urea, 7M thiourea, 4% CHAPS (w/v), 0.5% Triton X100, and 10 μL protease/phosphatase inhibitor cocktail. The dissolved samples were sonicated for 10 seconds at a constant duty cycle of 20%, with a 30 second interval (Ultrasonic Homogenizer 150V/T, Biologics Inc., Manassas, VA). The sonicated cell lysates were then placed on ice for one hour and were then centrifuged for 5 min at 14,000 x g. The supernatants containing soluble proteins were used for 2-DE analysis. The protein concentration was determined using the 2D Quant kit from GE Healthcare.

### Protein labeling, 2-DE electrophoresis, and MS analysis

To determine how protein changes may actually lead to the cytotoxic outcome and toxicity pathway alterations, we analyzed protein expression profiles in the treated BEAS-2B cells using difference in gel electrophoresis as previously described [15]. After the first and second gel electrophoresis, the resulting 2-DE gels were scanned using Typhoon 9410 scanner to produce digital images for protein quantitation analysis [15]. The differentially expressed proteins were picked up from Spro Ruby-stained SAD-PAGE gels, digested with trypsin, and analyzed using MS for protein identification [15]. Briefly, the gel dried plugs containing differentially expressed proteins were rehydrated using 10 μL of Proteomics Grade trypsin, and the digested and released protein peptides were desalted using C18 Zip tips (Millipore, Billerica, MA), and spotted onto a stainless steel MALDI plate for MALDI-MS/MS analysis. Proteins were identified using a 4800 MALDI TOF/TOF mass spectrometer (Applied Biosystems, Foster City, CA). MALDI-MS/MS sequencing and the final protein identification was done using Protein Pilot 3.0 software, searching against the human (Homo sapiens) sub-database of the SwissProt protein database (http://expasy.org/sprot/). Mass-selected MALDI-MS/MS spectra acquisition was performed to further increase protein sequence coverage. The reported proteins were identified based on a minimum of two unique MS/MS peptide sequence identifications characterized by the software confidence parameter of 95–99%.

### Pathway and network analysis

After protein identification, the accession numbers and fold changes of the differentially expressed proteins were imported into IPA to identify biological function, molecular function, canonical pathway, and tox list that are related to Ni (II)-induced toxicity at a molecular, cellular, and biochemical level. Each pathway or list was considered statistically significant when p < 0.05 after a Fisher Exact Test. In this method, the p-value for a given process annotation is calculated by considering the number of identified proteins that participate in that process and the total number of proteins that are known to be associated with that process in the selected reference set. The more identified proteins involved, the more likely the association is not due to random chance, and thus the more significant the p-value. IPA was also used to construct interaction networks of differentially expressed proteins identified by 2-DE.

### Multiplex ELISA for measurement of changes at expression and phosphorylation levels of the 14 pathway regulators

Extraction of total proteins from the control and Ni (II)—treated BEAS-2B cells, and ELISA assays using MSD Multi-Array kits were performed according to the manufacturer’s instructions (Meso Scale Discovery, Gaithersburg, MD). These multiplex ELISA assays allow simultaneous assessment of panels of functionally related proteins. The assays used included the Akt Signaling Assay panel (Cat. No. K1511D-1), which measures phosphorylated Akt (ser473), phosphorylated p70S6K (Thr421/Ser424) and GSK-3β (Ser9), the Human Proinflammatory Cytokine panel (Cat No. K15007B-1, which measures IL-6 and IL-8), the EGFR Family panel (Cat. No. K15106D-1), which measures EGFR, ErbB2 and IGF-1R), Apoptosis panel (Cat. No. K15106D-1), which measures Cleaved PARP (Asp214), Cleaved Caspase-3 (Asp175), Phospho-p53 (Ser15), Total p53), MAP Kinase Phosphoprotein panel (Cat. No. K15101A-3), which measures Phospho-p38 (Thr180/Tyr182), Phospho-ERK1/2 (Thr202/Tyr204; Thr185/Tyr187), Phospho-JNK (Thr183/Tyr185), and HIF-1α (Cat. No. C11DK-1). The ELISA assays were performed in triplicates. Briefly, 96-well plates were first blocked with the supplied blocking solution, washed either in supplied Tris wash buffer in phosphate buffered saline containing 0.05% Tween 20 (for cytokine assay only). The plates were blotted dry, and the extracted protein samples were then added. For all the ELISA assays, with the exception of the cytokine assay, protein samples were diluted in appropriate lysis buffer to the concentration of 0.4 or 0.8 μg/μL, depending on the assays. For the cytokine assay, the protein concentration ranged from 0.74 to 2.16 μg/μL. After the incubation with the corresponding primary antibodies that were localized in arrays at the bottom of the 96-wells, the wells were then washed and the corresponding secondary antibody solutions were then added. After the incubation with the secondary antibody, the wells were washed and the Read Buffer was added. The ELISA signals were recorded using the MSD Sector Imager 2400 and analyzed using Discovery Workbench 2006 software (Meso Scale Diagnostics, Gaithersburg, MD).

### ELISA for metallothionein-2 (MT-2)

The assay was performed according to the Manufacturer’s specifications (MyBiosource, San Diego, CA). One hundred microliter aliquots of MT-2 standards or samples were added to wells in a 96 well plate and incubated for 2 hr. The liquid from each well was removed and 100 μL of biotin antibody was added to each well and incubated for 1 hr at 37°C. An aspirator was used to remove liquid from the wells and the plate was washed three times using 200 μl of wash buffer. One hundred μl of HRP-avidin was then added to each well of the plate and the plate was incubated at 37°C for 1hr. After the incubation, the liquid from each well was aspirated and washed five times with wash buffer. TMB substrate (90 μL) was then added to each well and incubated for 20 minutes at 37°C in the dark. Finally, 50 μL of stop solution was added to each well and the plate was tapped to mix reagents. The optical density of each sample was determined sequentially at 450 and 570 nm. The optical readings at 570 nm was subtracted from the reading at 450 nm to remove the plate background reading The MT-2 expression level in each of the samples was extrapolated from the standard curve generated by the standards provided by the manufacturer.

### Statistical analysis

Data presented are expressed as mean ± SD. ELISA and 2-DE data obtained from triplicate assays were analyzed using a one-way ANOVA or a two-way comparison, as appropriate. For statistical analysis of ELISA data, multiple comparison corrections were performed for comparing the changes of 14 pathway regulators. The significance level applied to the comparisons of the entire family of pathway regulators was 95%. Statistical analysis were performed using GraphPad PRISM, version 5.0 (GraphPad Software, San Diego, CA), with p < 0.05 considered significant. For 2-DE gel electrophoresis, triplicate gels for each samples were run. Difference in protein expression levels in 2-DE gel spots were determined by DeCyder software. Student t test was used to evaluate difference in protein expression between control and treated experimental groups. Protein expression changes were considered to be significant when p < 0.05 and fold changes > 1.2.

## Results

### Overview of responses of cytotoxicity and 14 pathway regulating proteins to Ni (II) treatment

**Fig 1:**
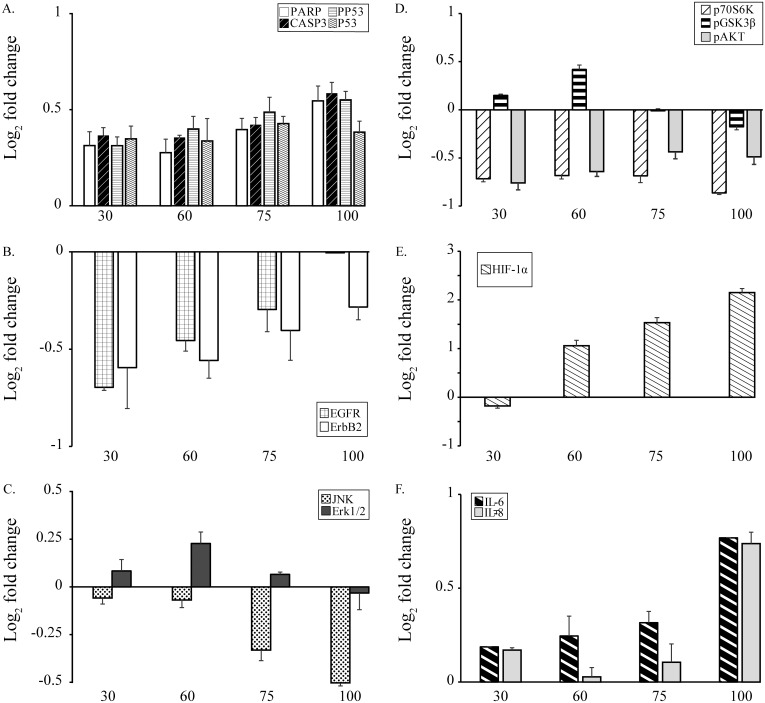
Changes in protein expression of the 14 pathway regulators induced by Ni (II). Panel A: Apoptosis panel proteins cleaved PARP, cleaved caspase-3, p53, pp53; Panel B: Two cell growth panel proteins EGFR and ErbB2; Panel C: MAP Kinase panel proteins JNK and Erk1/2; Panel D: phosphorylated p70S6K, GSK3β, and Akt; Panel E: HIF-1α; Panel F: IL-6 and IL-8. X-axis is Ni (II) concentrations (μM). Y-axis: log_2_ relative-fold change of expression and/or phosphorylation levels compared to untreated controls measured using multiplexed ELISA. Protein significantly altered over the corresponding controls were identified and plotted as up-regulated (above the X-axis) or down-regulated (blow the X-axis). Statistical differences in protein expression or phosphorylation between Ni (II) treated and control group were determined by Student’s t-test (n = 3, p < 0.05).

Exposure of BEAS-2B cells to 30, 60, 75, and 100 μM Ni (II) resulted in relative survivals of 0.94, 0.88, 0.73, and 0.52, and were classified as very low, low, moderate, and high toxicity exposures, respectively. All of the 14 pathway regulators examined were altered at the level of expression or phosphorylation in BEAS-2B cells treated with Ni (II) at each of the concentrations tested, with changes ranging from Log_2_−1.2 to 2.2-fold (Fig 1). The down-regulated proteins included EGFR, ErbB2, AKT, GSK3β, p70S6K, Erk1/2, and JNK. The up-regulated proteins include cleaved-PARP, cleaved caspase-3, HIF-1α, p53, phosphorylated p53, and cytokines IL-6 and IL-8. In addition to the cytotoxicity and protein changes observed, apoptotic cell death also increased with increasing Ni (II) concentrations (Fig A in S1 File). Increased frequencies of apoptotic nuclei were observed in the treated BEAS-2B cells with low-, moderate-, and high-toxicity levels.

### Hierarchical clustering analysis of protein responses to Ni (II) at different concentrations

**Fig 2:**
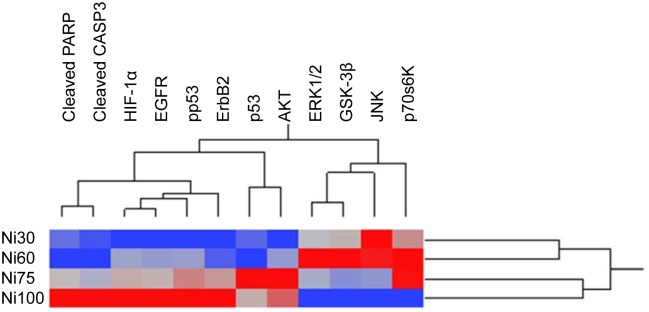
Hierarchical clustering of 12 differentially expressed or phosphorylated proteins in BEAS-2B cells treated with Ni (II). Dendrogam was obtained from hierarchical cluster analysis which is mainly based on dynamic changes of proteins. Blue fields indicate down-regulation and the red fields indicate up-regulation of the proteins. Proteins with most nearly identical dynamic pattern and similar functions are usually clustered together.

To determine how the change in expression or phosphorylation of a protein was related to the change in that of another protein, an unbiased multivariate clustering method was used to correlate 12 non-cytokine pathway regulator protein responses in BEAS-2B cells treated with Ni (II) at 4 different concentrations (Fig 2). The 12 proteins were cluster-ordered on the basis of their expression or phosphorylation changes across BEAS-2B cells treated with Ni (II) at 4 doses. The dynamics changes of proteins with most nearly identical pattern appear side by side on the x-ordinate. The 4 doses of Ni (II) with most identical protein expression or phosphorylation patterns appear side by side on the y-co-ordinate. These pathway regulating proteins were grouped into 4 clusters. Within each of the clusters identified, the members responded more similarly to the different concentrations of nickel than did members of the other clusters. The clusters identified were 1) cleaved PARP and cleaved CASP3; 2) HIF-1α, pp53, EGFR, and ErbB2; 3) p53 and phosphorylated Akt; and 4) JNK, ERK1/2, GSK-3β, and p70S6K. The functionally-related proteins were generally grouped into the same cluster, suggesting co-regulation at the level of toxicological pathways or processes. In addition to the finding that functionally related proteins were grouped into the same clusters, we also observed that the overall changes in protein response differed between Ni (II) treatments resulting in lower cytotoxicity (30 and 60 μM), and those inducing higher cytotoxicity (75 and 100 μM) (Fig 2). This observed correlation between protein changes and cytotoxicity identifies a tipping point in pathway activation associated with induction of cytotoxic responses.

### Correlations of protein responses with Ni (II) induced cytotoxicity

**Fig 3:**
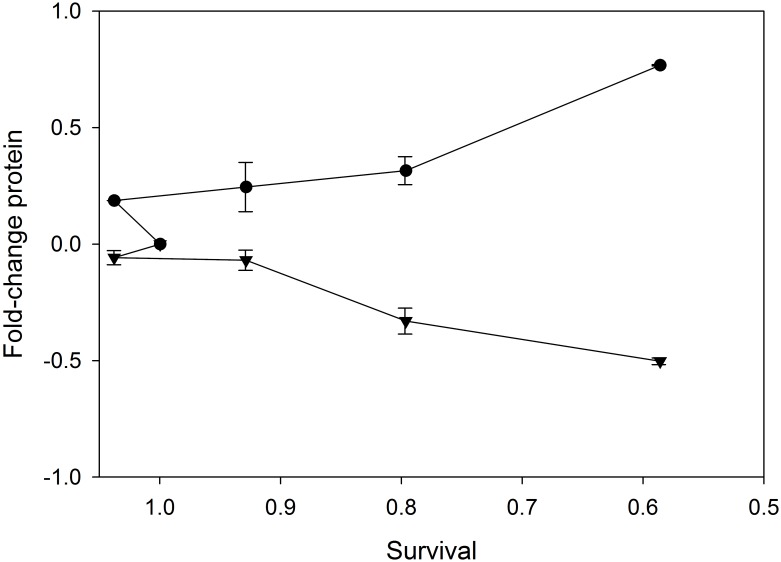
Relative cell survival (X-axis) vs. protein expression or phosphorylation levels (Y-axis) in BEAS-2B control cells treated with Ni (II) at 4 different concentrations. ●: IL-6; ▼: JNK; A good linear correlation was observed between IL-6 expression levels and cell survival rates. Similar linear correlation was observed between JNK phosphorylation levels and cell survival rates (n = 3, p < 0.05).

As shown in Fig 1, dynamic and diverse protein responses (either changes at protein expression or phosphorylation level) related to cytotoxicity were observed in the cells exposed to Ni (II) at 4 doses. HIF-1α showed the greatest range of expression levels with increased concentration of Ni (II). Cytotoxicity decreased with increasing phosphorylation levels of the MAP kinase JNK, but increased with the increasing expression levels of IL-6 and HIF-1α. Some of changes at either expression levels such as IL-6 or phosphorylation levels such as JNK are monotonically related to cell survival in BEAS-2B cells treated with Ni (II) (Fig 3). The collinearity between the protein changes with the cytotoxic responses of BEAS-2B suggests that this small set of signaling proteins such as IL-6 and JNK has a critical role in regulating downstream toxicity pathways and determining cytotoxicity of BEAS-2B cells treated with Ni (II).

### Identification of differentially expressed proteins in BEAS-2B cells treated with Ni (II) using 2-DE

**Fig 4:**
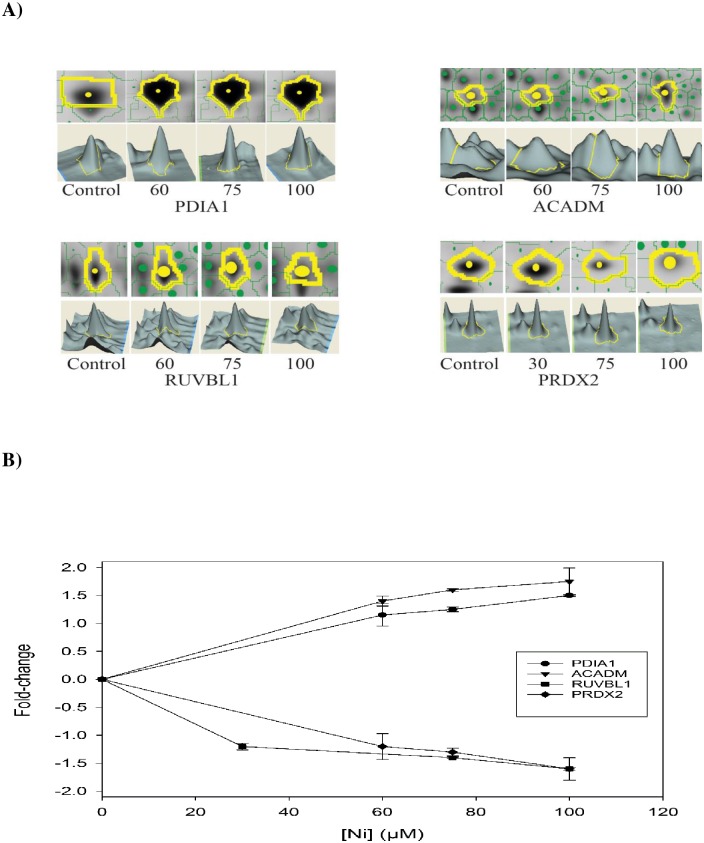
Four representative proteins, PDIA1, ACADM, RUVBL1, PRDX2 identified using 2-DE profiling were either increased or decreased in a concentration responsive manner. A) The upper panel is an expanded view of 2-DE gel images for each protein spot, and the lower panel is a 3D view of the protein spot. The volume of each spot was calculated using Decyde software and is graphically represented. The expression level of the protein is proportional to the volume of the peak. B) The line graphs indicate the dose-responsive changes of proteins in BEAS-2B cells treated with 4 different concentrations of Ni (II). Statistical differences in expression of these proteins between Ni (II) treated and control group were determined by Student t-test (n = 3, p < 0.05).

In addition to the analysis of responses of 14 key signaling proteins regulating metal toxicity pathways, we also investigated novel changes at expression levels of the downstream proteins in BEAS-2B cells treated with Ni (II) at four different concentrations using 2-DE and mass spectrometry. A total of 249 proteins were found to be significantly (*p* <0.05) altered, and 128 proteins were identified by MS from BEAS-2B cells treated with Ni (II) at four different concentrations (Fig B and Table A in S1 File). Of the identified proteins, 52 proteins were up-regulated and 77 proteins were down-regulated. Thirty seven proteins were detected from BEAS-2B cells treated with Ni (II) at three concentrations. Of the 37 proteins that showed concentration-response relationships, 15 proteins were up-regulated and 22 were down-regulated. These proteins included down-regulated oxidative stress response proteins, such as peroxiredoxins 2 (PRDX-2) and DNA stimulated ATPase activity such as RuvB-like 1(RUVBL1). Upregulated proteins include protein disulfide isomerase (PDIA1) and medium chain specific acyl-coA dehydrogenase (ACADM) (Fig 4).

### Mapping differentially expressed proteins to canonical pathways and tox lists

**Fig 5:**
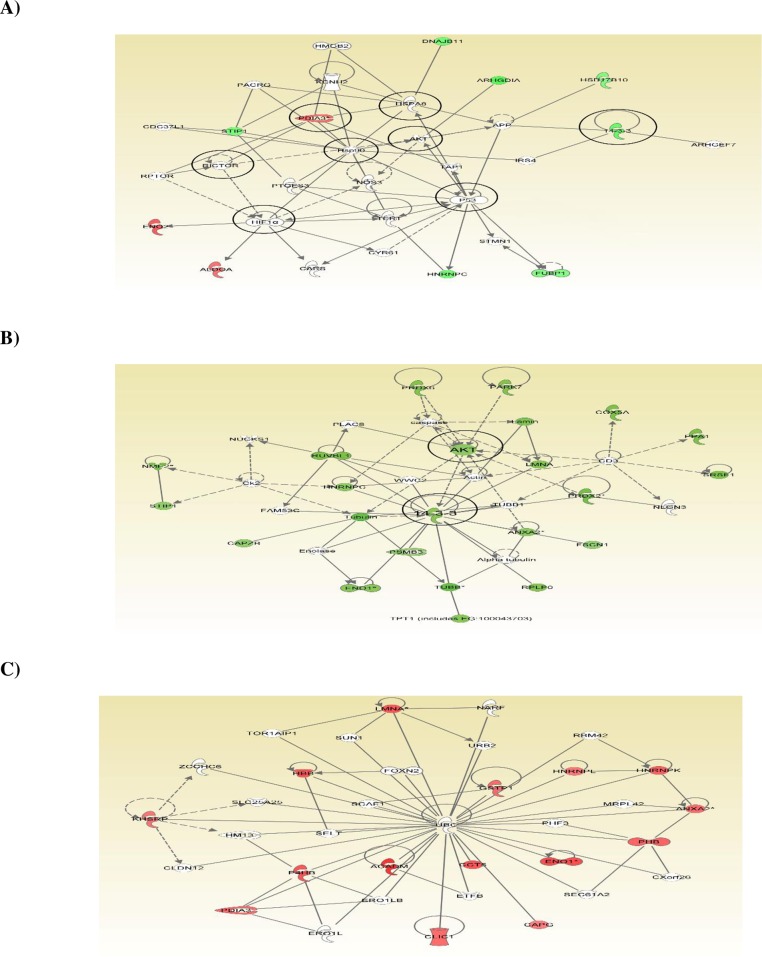
Networks of proteins showing inter-relationships and pathways which was obtained using IPA. A) A sub-network of some differentially expressed proteins identified from BEAS-2B cells treated with 100 μM of Ni (II). Green fields indicate down-regulation and the red fields indicate up-regulation of the differentially expressed proteins. B) Network of the proteins altered at multiple concentrations that were down-regulated. C) Network of the proteins altered at multiple concentrations that were up-regulated. For protein network or pathways analysis, statistical significance was determined with Fisher’s exact test (p < 0.05).

IPA software was applied to map 128 differentially expressed proteins identified from BEAS-2B cells treated with Ni (II) at 30, 60, 75, and 100 μM to predefined canonical pathways and toxicity lists to uncover pathways altered by Ni (II). Fig 5A is a representative protein network of inter-relationships identified from BEAS-2B cells treated with 100 μM of Ni (II). IPA analysis of 67 differentially expressed proteins identified from BEAS-2B cells treated with 100 μM of Ni (II) identified three protein networks, one of which is shown in Fig 5A. Proteins in red or in green colors in the network were up-regulated or down-regulated respectively in the treated BEAS-2B cells. This network displays two major subnetworks, a P53 subnetwork that connects to a second subnetwork of HIF-1α. In addition to P53 and HIF-1α, there are other 6 smaller hubs that connect differentially expressed proteins: Akt, heat shock protein A8 (HSPA8), heat shock protein 90 (HSP90), protein disulfide isomerase A3 (PD1A3), regulatory associated protein of mTOR complex 1 (RICTOR), and 14-3-3 (YWHAG), which are marked by circles. The general functions of these pathways identified from the BEAS-2B cells treated with Ni (II) at 100 μM include regulation of apoptosis, hypoxia, glycolysis and glycogenesis, cell growth signaling, inflammation, and oxidative stress. These results are consistent with those from ELISA analysis of the expression and phosphorylation of 14 regulating proteins, in which p53, pp53, HIF-1α, and Akt were all differentially modified and played critical roles in determining NI(II) cytotoxicity.

In addition, we also compared IPA analysis results obtained from Ni (II) at 4 different concentrations in order to determine protein and pathway changes in response to different doses of Ni (II), and to identify potential toxicity pathways altered by Ni (II). We hypothesized that the pathways modified at multiple concentrations of Ni (II) are more important in driving cytotoxicity than pathways that were altered only by a single concentration. Many of these same pathways have been identified as being altered in various diseases and disorders, molecular and cell functions, and canonical pathways, and IPA tox lists, as shown in Table B in S1 File. Cancer is the most significantly associated disease identified by IPA analysis, which is consistent with nickel being a potent human carcinogen. Cell death and small molecular biochemistry are the two most affected molecular and cell functions identified. Canonical pathway and tox list analyses indicated mitochondrial dysfunction, glutathione metabolism, glycolysis and gluconeogenesis, hypoxia inducible factor signaling, and oxidative stress are the most common and important pathways affected by Ni (II) at different doses.

Fig 5B shows the inter-relationships of the 23 downregulated proteins that were altered at multiple doses. These proteins are interconnected by Akt and 14-3-3 hubs to form protein clusters of pathways, suggesting the importance of the down-regulation or inhibition of Akt and 14-3-3 pathways in Ni (II)-mediated toxicity. The down-regulation of Akt suggested by IPA analysis were consistent with the results obtained from ELISA analysis of the 14 regulating proteins in which Akt panel proteins were down-regulated. In addition to the pathway analysis of the proteins downregulated at multiple doses, we also analyzed protein networks of the up-regulated proteins that were altered at multiple doses using IPA. As shown in Fig 5C, these upregulated proteins are inter-connected and centered by ubiquitin C (UBC), suggesting that the protein degradation mediated by ubiquitination is a key pathway involved in cytotoxic responses to Ni (II).

## Discussion

In the present study, the overall protein and pathway responses to nickel treatments were integrated across the individual proteome changes obtained from BEAS-2B cells treated with Ni (II) at both low concentrations i.e. 30 and 60 μM, and high concentrations i.e. 75 and 100 μM. The different doses were selected based on preliminary cytotoxicity assays in order to establish quantitative relationships between the changes at the protein expression and phosphorylation levels and cytotoxicity. Proteomic analysis of BEAS-2B cells treated with relatively high concentrations, such as 75 and 100 μM of Ni (II), could help to identify toxic modes of action of Ni (II), since the higher concentrations of Ni (II) provide additional sensitivity that help in the initial identification of significant effects of Ni (II) on both protein and cytotoxic responses. The relatively lower concentrations of Ni (II) treatments, such as 30 and 60 μM, could help to confirm whether the high concentration observations were relevant with those at the lower concentrations. It was our hypothesis that the consistent proteome alterations, including expression and phosphorylation changes, and alteration in patterns or profiles obtained from BEAS-2B cells treated with multiple concentrations of Ni (II) would identify those protein changes that were critical for defining Ni (II) toxicity. The Ni (II)-specific toxicity pathway regulators were identified by investigating the expression and phosphorylation changes of 14 upstream pathway regulating proteins using ELISA, and NI (II)-induced toxicity pathways were identified by IPA analyses of the downstream differentially expressed proteins using 2-DE in one experimental system BEAS-2B cells.

We initially investigated the expression or phosphorylation levels of 14 metal toxicity pathway regulators in BEAS-2B cells treated with Ni (II) at 4 different concentrations using ELISA. Our results identified dynamic changes of these pathway regulators as the promising key and Ni (II)-specific signaling proteins regulating Ni (II)-induced ecotoxicity such as HIF-1α and IL-6. These pathway regulators were altered with the changes of Ni (II) concentrations in a dose-responsive manner (Fig 1). In addition, the expression or phosphorylation levels of these pathway regulators were correlated with Ni (II) cytotoxicity data for identification of a small group of proteins or pathway regulators which are critical or even determine the toxicity of Ni (II) to BEAS-2B cells (Fig 3). In addition to identifying these individual, specific, and critical pathway regulators of Ni (II)-induced toxicity, 2-DE analyses of BEAE-2B cells treated with Ni (II) at low and high toxicity of Ni (II) also identified a large numbers of differentially expressed proteins involved in Ni (II)-induced cytotoxicity. Due to differences in protein abundances and technical limits, the Ni (II)-specific toxicity pathway regulators such as Akt and HIF-1α were not detected in 2-DE analyses. However, IPA analyses of the networks or pathways of the differentially expressed proteins displayed that many of these pathway regulators such as Akt and HIF-1α served as the major hubs of the protein networks and connected the differentially expressed proteins identified using 2-DE. Therefore, it is reasonable to assume that these Ni (II)-specific pathway regulators identified using ELISA and the toxicity pathways identified using 2-DE are critical to Ni (II)-induced toxicity responses in BEAS-2B cells. The detailed description, integration, and validation of the effects of these proteins and pathway responses on Ni (II)-induced cytotoxicity responses will be reported in a series of papers in preparation. This paper is the first comprehensive proteomic report integrating the up-stream and downstream protein responses identified by ELISA and 2-DE and the cytotoxic data obtained from multiple Ni (II) treatments of BEAS-2B cell for the identification of the core proteins and pathways underlying and even determining Ni (II)-induced toxicity responses. Based on the findings from the present study, the core protein responses and pathways that lead to deleterious cellular effects of Ni (II) could be classified into four general categories including glycolysis and glycogenesis, apoptosis, MAP Kinase mediated-stress response pathways, and ubiquitin-mediated protein degradation.

### Alteration of glycolysis and gluconeogenesis pathways by HIF-1α and Akt panel proteins

HIF-1α is an oxygen-dependent transcriptional activator, and acts as a master regulator of numerous hypoxia-inducible genes under hypoxic conditions [16]. The targeted genes and proteins of HIF-1α are especially related to cell proliferation and survival, and glucose metabolism [17]. HIF-1α inductions by Ni compounds has been described previously [10]. In the present study HIF-1α displayed the greatest dynamic range of changes in protein expression level in response to Ni (II) treatment as compared to all the other pathway regulating proteins examined in this study. HIF-1α expression in BEAS-2B cells increased with increasing concentration of Ni (II (Fig 1). Correspondingly, many downstream functional proteins involved in HIF-1α-mediated glucose metabolism, such as proteins ALDA, Eno-1 and -2, were significantly upregulated in BEAS-2B cells treated with Ni (II) at different concentrations. This suggests that the observed Ni (II) induction of HIF-1α transcription factor thereby regulated the expression of glycolytic enzymes and proteins involved in glucose metabolism.

Akt panel proteins, including phosphorylated Akt, p70S6K, and GSK3β, are the major regulators of glucose metabolism especially glycogenesis pathways [18]. The phosphorylation levels of these proteins were all significantly decreased (Fig 1). Our ELISA results showed that the phosphorylation level of p70S6K in BEAS-2B cells treated with Ni (II) at 30, 60, 75, and 100 μM was decreased to –0.72, -0.68, -0.68, and –0.87 fold of that in control cells with the increased concentrations of Ni (II). Proteins involved in glucose and glycogenesis pathways, such as eukaryotic translation elongation factor 2 (EF-2) and protein 14-3-3, that are regulated by p70S6K, were significantly decreased as well [Table A in S1 File]. GSK3β, which is known to phosphorylate and thus inactivate glycogen synthase [19] was also significantly downregulated in BEAS-2B cells treated with 75 and 100 μM of Ni (II). The down regulation of phosphorylation of the three Akt panel proteins, Akt, p70S6K, and GSK3β, strongly suggests their roles in the inhibition of glycolysis and activation of gluconeogenesis pathways in BEAS-2B cells treated with Ni (II). This suggestion was also supported by the alterations of some proteins that tend to favor gluconeogenesis, such as 3-hydroxyaxylcoenzyme A dehydrogenase (HADH) [20].

Alterations of the upstream pathway regulators and downstream functional proteins and enzymes of glycolysis and gluconeogenesis pathways are expected to affect cellular energy metabolism, and consequently influence oxidative phosphorylation rates and ATP levels, and eventually cell survival or death.

### Activation of apoptosis proteins and pathways in response to Ni (II) treatments

In order to determine if apoptosis was responsible, at least partially for cell death, we performed ELISA assays on a panel of apoptosis markers including p53, pp53, cleaved caspase 3, and cleaved PARP (Fig 1). Additional methods used include immunostaining of apoptotic cells using a high content assay (HCA) (Fig A in S1 File). Both HCA and ELISA results (Fig 1 and Fig A in S1 File) demonstrated an increase in apoptosis with increased concentrations of Ni (II). In addition, some downstream proteins involved in apoptosis were also altered, based on the 2-DE analysis of BEAS-2B cells treated with Ni (II). It is well-recognized that during apoptosis, the cell undergoes dramatic changes in morphology due to a complete reorganization of the cytoskeleton [21]. Interestingly, the basic components of the cytoskeleton in eukaryotic cells including intermediate filaments such as keratin (KRT) and vimentin (VIM), actin (ACT) and microfilaments, and microtubulins such as tubulin (TUBB) in BEAS-2B cells treated with nickel were also altered. These results suggest that the subcellular rearrangement of cytoskeletal proteins and possible phenotype changes of the BEAS-2B cells were due in part to apoptosis induced by Ni (II). 14-3-3 family members are dimeric phosphoserine-binding proteins that participate in signal transduction and checkpoint control pathways [22]. It is known that the 14-3-3 proteins have a major function in the control of apoptosis [23]. However, the role of 14-3-3 proteins in Ni (II) induced apoptosis and toxicity has remained uncharacterized. In the present study 14-3-3 proteins were down-regulated in BEAS-2B cells treated with Ni (II), and the decreased expressions of 14-3-3 proteins might affect cytoskeletal structure stability and promote Ni-mediated apoptosis as well. In addition, the results from the protein networks and toxicity pathway analysis of Ni (II)-treated BEAS-2B cells demonstrated that mitochondrial membranes in Ni (II)-treated BEAS-2B cells were damaged and dysfunctional (Table B in S1 File), which is an important indicator of apoptosis in the BEAS-2B cells.

### Activation of MAP Kinases and stress response pathways

It has been reported that metals trigger a signal cascade that begins with the activation of members of the MAP kinase family which includes JNK and Erk1/2 [24]. In the present study, JNK was downregulated and Erk1/2 was up-regulated in BEAS-2B cells treated with 30 μM Ni (II), whereas at higher concentrations, resulted in significant cell death, with both JNK and Erk1/2 down-regulated in a concentration-dependent manner. This cytotoxic response is associated with the inhibition of JNK and Erk1/2. JNK and Erk1/2 regulate a wide variety of key biochemical pathways and cellular processes such as oxidative stress, cell proliferation and differentiation, cell cycle and growth, apoptosis, inflammation, and other stress responses [1, 25]. Nickel ions are haptens which can penetrate the skin barrier and interact with carrier molecules (usually proteins) to become immunogenic [26] Previous studies showed that a direct cytotoxic effect of nickel on cultured human proliferative keratinocytes is accompanied by an increased production of pro-inflammatory signal proteins such as TNFα [27] and JNK and Erk1/2 are critical to TNFαinduced IL-6 and IL-8 expression and production [28]. The ability of this family of MAP kinases to induce stress response pathways raised the questions as to how MAP kinase phosphorylation is altered resulting in inflammation response to Ni (II) treatments in human BEAS-2B cells in which JNK and Erk1/2 were differentially regulated. As shown in Fig 1, IL-6, a traditional marker of inflammation that promotes the induction of acute phase proteins [29] and IL-8, a recruiter for neutrophils and macrophages to the site of inflammation [30] were up-regulated in BEAS-2B cells treated with Ni (II) at four different concentrations (Fig 1). These two cytokines may initiate a basic and common inflammation pathway or mechanism through which BEAS-2B cells responded to Ni (II). The consistent down-regulation of JNK and Erk1/2 suggested that the activations of IL-6 and IL-8 were not through MAP Kinase family members in Ni (II) treated BEAS-2B cells. It has been reported that annexin 2A (ANXA2) expression is highly up-regulated in many types of cancer, and the molecular target for Ni (II) toxicity [31, 32]. ANXA2 is involved in NFκB signaling pathways, and increase the transcriptional activity of NFκB in both the resting and activated states and upregulated the transcription of several target genes downstream of NFκB, including that encoding IL-6 [33]. The consistent up-regulation of ANXA2 in BEAS-2B cells treated with Ni (II) at four concentrations suggested that it may play important roles in the activation of IL-6, IL-8, and the related inflammation responses.

It is known that cells defend themselves against ROS by elaborate systems of biological defense [34]. Ni (II)-induced oxidative stress either up-regulated or down-regulated antioxidant pathways, depending the concentrations of Ni (II). Two major anti-oxidant defense systems mediated by MAP Kinases including peroxiredoxin family members (PRDXs) and metallothionein (MT) [35] were significantly altered. PRDXs are a group of peroxidases that reduce H_2_O_2_ with concomitant oxidation of active site cysteine residues [36]. These proteins protect against the damaging effects of ROS or in a signaling role by controlling levels of H_2_O_2_. Mammalian PRDXs are highly expressed in the cytoplasm of cells, where they are known to have multiple anti-oxidant functions [36]. In BEAS-2B cells treated with Ni (II) at different concentrations, PRDX family members were differentially expressed. Generally, PRDXs were usually up-regulated at low concentrations of Ni (II) and down-regulated at higher concentrations.

The induction of metallothionein (MT) has been reported to be critical in limiting nickel-induced lung injury in intact mice [37]. Metallothionein-2 (MT-2) is a heavy metal-binding protein that participates in a variety of protective stress responses, such as limiting nickel-induced lung injury in mice by scavenging hydroxyl radical [38]. In the present study, the expression level of MT-2 in BEAS-2B cells treated with 30 or 75 μM was significantly increased (Fig C in S1 File), which suggested that MT-2 played some role in the protection of Ni (II)-induced oxidative stress and toxicity

### Protein degradation in BEAS-2B cells treated with Ni (II)

One protein, which was highly connected to multiple proteins altered at all concentrations of Ni (II) tested, was an ubiquitin protein UBC (Fig 5C). All of these proteins connected to UBC were up-regulated at all concentrations of Ni (II). Most of these proteins had not previously been linked to Ni (II)-induced effects. The ubiquitin-proteasome pathways are the major proteolytic systems in the cytosol of eukaryotic cells, catalyzing the selective degradation of short-lived proteins and the rapid elimination of proteins with abnormal conformation [39–40]. UBC has been shown to be involved in regulation of various biological processes such as the cell cycle, apoptosis, and inflammation, which are contributors to human cell toxicity and carcinogenicity [39–40]. While the effects of the altered expression of ubiquitin proteins by Ni (II) on overall protein degradation or on specific proteins are not known, it is clear that the ubiquitin proteins and pathways were important events in Ni (II)-induced cytotoxicity.

### Proteins and pathways determining the cytotoxic responses of BEAS-2B cells to Ni (II)

**Fig 6:**
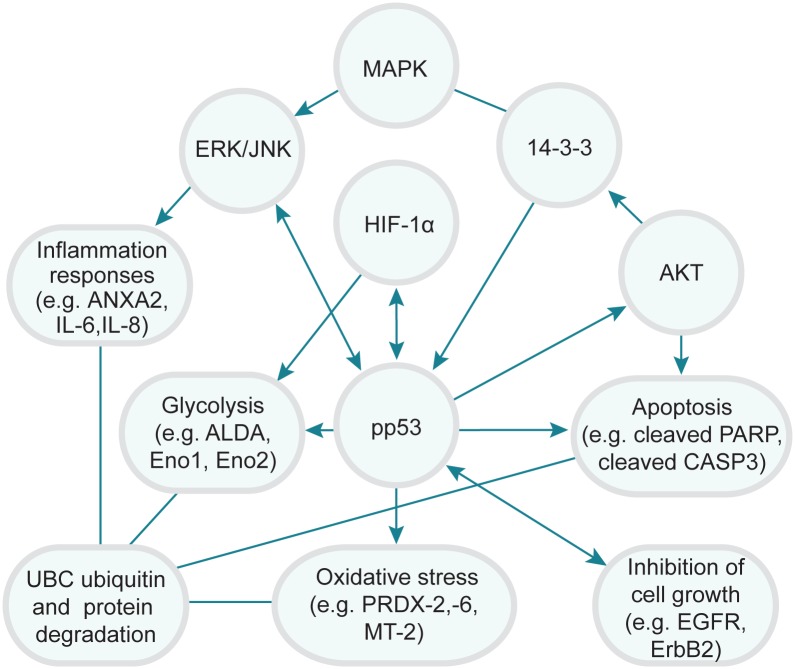
Schematic representation of the interplay of the core proteins and cytotoxicity pathways mediated by Ni (II). Key proteins or pathways that were likely to play critical roles in determining sensitivity or cytotoxic response of BES-2B cells to Ni (II) treatments are shown here.

Generation of toxicity and even induction of cell death in BEAS-2B cells treated with Ni (II) is a multiple-step process with a wide variety of molecular changes especially protein changes leading to the adverse outcomes or phenotype changes. Various molecular determinants of toxic effect of Ni (II) have been elucidated in BEAS- 2B cells and other biological systems [6, 9]. However, the key molecular factors and pathways that are critical or even determine adverse outcomes or phenotypes of nickel still remain to be elucidated. For this purpose, we analyzed 14 toxicity pathway regulators and 128 downstream functions proteins to identify crucial proteins and pathways that are critical or even determine toxicity responses of BEAS-2B cells to Ni (II) treatment. Two approaches were used to reach this goal. First, we correlated BEAS-2B cell survival rates with protein expression and phosphorylation levels. As shown in Fig 3, changes in expression/phosphorylation levels of IL-6 and JNK are monotonically related with cell survival in BEAS-2B cells treated with Ni (II) concentrations, suggesting that these proteins are likely the ones conferring or determining toxicity after Ni (II) treatments. These proteins were likely to be the major components of the core toxicity pathways of Ni (II). Secondly, 2-DE protein profiling of Ni (II) treated BEAS-2B cells was used to identify downstream functional proteins potentially relevant for cytotoxic responses to Ni (II). Four predominant toxicity pathways in response to different doses of Ni (II) were identified such as HIF-1α and Akt panel protein-mediated glycolysis and gluconeogenesis pathways, MAP Kinase-mediated inflammation and oxidative responses, cleaved PARP and cleaved caspase-3 mediated apoptosis, and UBC-mediated protein degradation using IPA. Interestingly, the major toxicity pathways identified by 2-DE profiling were consistent with those obtained from ELISA and the protein and cytotoxicity correlation studies. For example, HIF-1α was found to be activated and increased in a dose-response manner based on the ELISA measurement results. Consistent with the activation of this transcription factor, a number of HIF-1α inducible proteins involved in glycolysis, such as Eno 1, Eno 2 and ALDA, were also significantly up-regulated, based on the 2-DE protein expression profiling results. Therefore, these corresponding pathways, linked to upstream protein determinants HIF-1α, pp53, ErbB2, IL-6, and JNK, were likely to be central pathways of Ni (II) toxicity. Taken together, we proposed a core protein and toxicity pathway map (Fig 6) to describe the interplay of these proteins and pathways determining the cytotoxic responses of BEAS-2B cells to Ni (II). The diagram was generated based on the quantitative correlations between the expression and phosphorylation levels of these proteins with cell survival rates, and the connectedness of these proteins as hub nodes in the networks of BEAS-2B cells treated with Ni (II) at four different concentrations using IPA. In these protein sub-networks, as well as merged networks, HIF-1α, pp53, JNK, 14-3-3 and AKT were usually found to be the major hubs, interacting with many of the downstream functional proteins identified using 2-DE based protein profiling. The IPA analysis further confirmed the crucial roles of these key proteins and pathways in determining cytotoxic responses of BEAS-2B cells to Ni (II).

## Conclusions

In summary, this integrated proteomic approach used in the present study identified key proteins and toxicity pathways that were modulated in the cellular response to Ni (II) exposure, and the likely determinants of nickel-induced cellular toxicity. This small set of key proteins were mainly involved in glycolysis and gluconeogenesis pathways, apoptosis, protein degradation, and stress responses including inflammation and oxidative stress. Thus, the identification of key pathways involved in specific toxicity response enables the use of such proteomic signatures as predictive tools to facilitate toxicity screening of nickel and other chemicals.

## Supporting Information

S1 File Fig A: Representative fluorescence photographs of BEAS-2B cells stained with Hoechst (upper-panel) and Phalloidin (lower panel) dyes. Fig B: Representative 2-DE gel images of control and BEAS-2B cells treated with 100 μM of Ni (II). Fig C: Ni (II) increased MT-2 expression in BEAS-2B cells treated with 30 μM and 75 μM of Ni (II). Table A: List of the differentially expressed pro-teins identified from BEAS-2B cells treated with Ni (II) at 4 concentrations. Included are all pro-teins altered by at least 1.2 fold in expression and detected with a FDR of less than 0.15 to elimi-nate false positive proteins. Table B: Comparisons of the top diseases and disorders, molecular and cell functions, canonical pathways and toxlists in BEAS-2B cells treated with 30, 60, 75 and 100 μM of Ni (II).
(DOCX)

## Abbreviations

TUB: Tubulin
HIF-1α: Hypoxia inducible factor alpha
NFκB: Nuclear factor NFKappa B
IL-8: Interleukin 6 (IL-6); Interleukin 8
CLIC1: Chloride intracellular channel protein 1
RUVBL1: RuvB-like protein 1
HNRNPC: Heteronuclear ribonucleoprotein C
PPA1: Inorganic pyrophosphatase 1
ALD: A Aldolase A
PRDX2: Peroxiredoxin 2
PRDX6: Peroxiredoxin 6
ENO1: Enolase 1
ENO2: Enolase 2
VIM: Vimentin
ACT: Actin
HADH: Hydroxyaxylcoenzyme A
UBC: ubiquitin C
KRT: Keratin
ACADM: Medium chain specific acylcoA dehydrogenase
PDIA1: Protein disulfide isomerase A1
EF2: Elongation factor 2
GSK3β: Glycogen synthase kinase 3β
