# Supplementary material for: Proteomic Assessment of Biochemical Pathways That Are Critical to Nickel-Induced Toxicity Responses in Human Epithelial Cells
Source: PLoS One. 2016 Sep 14;11(9):e0162522. doi: 10.1371/journal.pone.0162522 (PMC5023113; doi:10.1371/journal.pone.0162522)
Supplement: S1 File — Fig A: Representative fluorescence photographs of BEAS-2B cells stained with Hoechst (upper-panel) and Phalloidin (lower panel) dyes. Fig B: Representative 2-DE gel images of control and BEAS-2B cells treated with 100 μM of Ni (II). Fig C: Ni (II) increased MT-2 expression in BEAS-2B cells treated with 30 μM and 75 μM of Ni (II). Table A: List of the differentially expressed pro-teins identified from BEAS-2B cells treated with Ni (II) at 4 concentrations. Included are all pro-teins altered by at least 1.2 fold in expression and detected with a FDR of less than 0.15 to elimi-nate false positive proteins. Table B: Comparisons of the top diseases and disorders, molecular and cell functions, canonical pathways and toxlists in BEAS-2B cells treated with 30, 60, 75 and 100 μM of Ni (II). (DOCX) [file pone.0162522.s001.docx]

**S1 File:**

**Fig A: Representative fluorescence photographs of BEAS-2B cells stained with Hoechst (upper-panel) and Phalloidin (lower panel) dyes.** The Hoechst dye stained morphological normal nuclei dimly blue, whereas apoptotic nuclei demonstrated condensed, intensively bright blue, and smaller nuclei. Staining of the cells with Phalloidin showed that controls as well as non-toxic cells exhibited a well-defined F-actin network that was mainly organized into stress fibers. Apoptotic nuclei were marked by arrows. The BEAS-2B cells treated with 30, 60, 75 and 100 µM of Ni (II) showed non-, low-, moderate-, or high-toxicity, respectively. Condensation of nucleus and disruption of the actin microfilament network were observed in these cells, and increased with the increases of Ni (II) toxicities. Apoptotic nucleus and disruption of actin microfilament were not observed in negative control cells and BEAS-2B cells. BEAS-2B cells treated with taxol were used as positive controls.


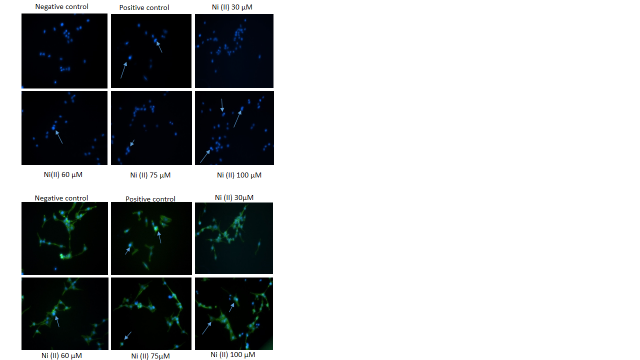


**Fig B: Representative 2-DE gel images of control and BEAS-2B cells treated with 100 µM of Ni (II).** Fifty microgram if each protein sample was subject to 2-DE. Spot numbers of proteins in control and the treated gel images are identical.

**
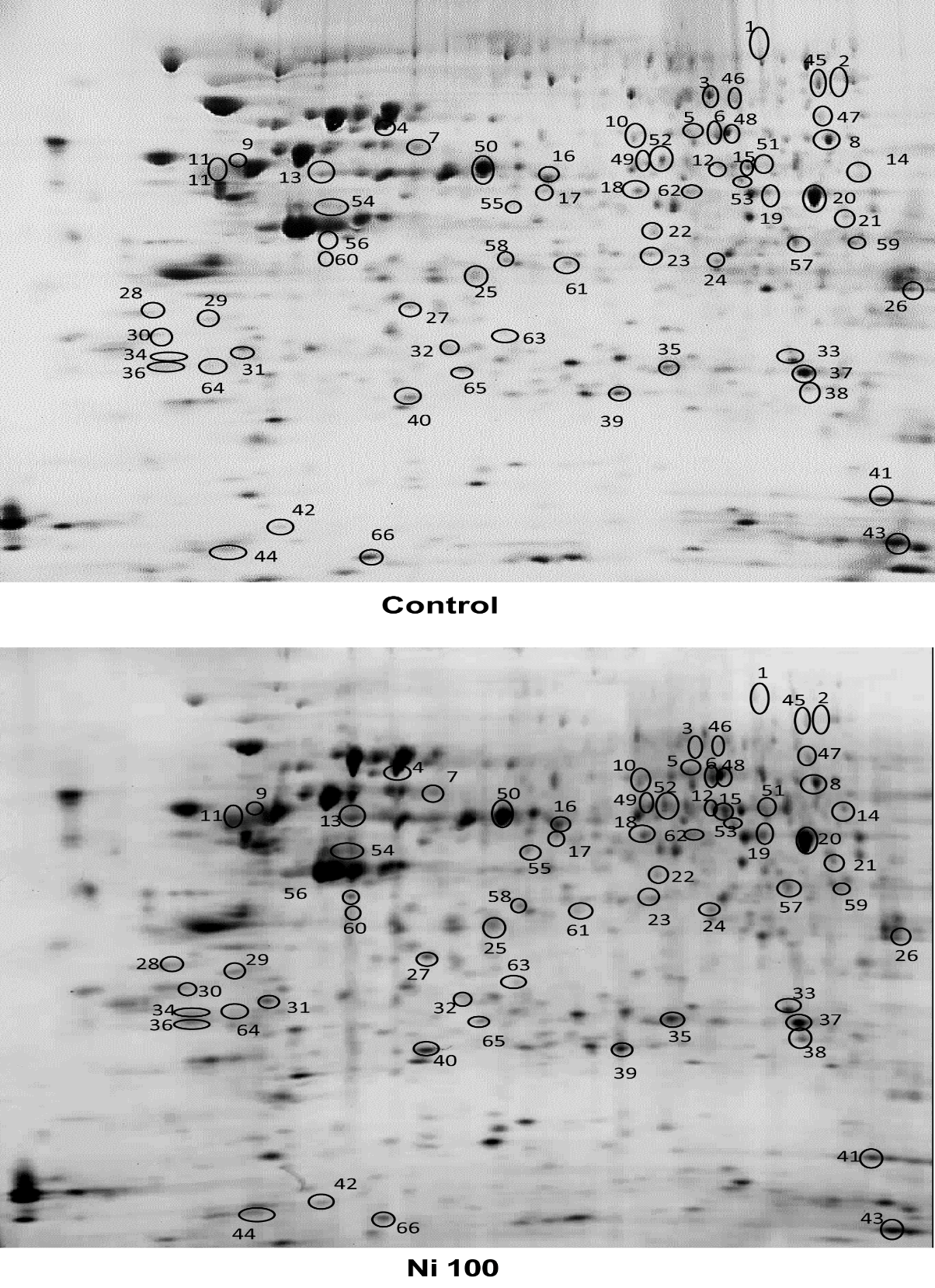
**

**Fig C**: **Ni (II) increased metallothionein-2 (MT-2) activity.** The MT-2 activity in BEAS-2B cells treated with 30 µM and 75 µM was significantly increased 1.25-fold as compared with the control. Data represent mean + STD (n= 3, p < 0.05).





**Table A**: **List of the differentially expressed proteins identified from BEAS-2B cells treated with Ni (II) at 4 concentrations.**

| Sample ID (30µM) | Abbrev | Protein ID | Fold  change | T-test | Accession No | pI | MW(kDa) | Matched peptides | % Coverage | P value |
| --- | --- | --- | --- | --- | --- | --- | --- | --- | --- | --- |
| 1 | TUBB | Tubulin chain | -1.2 | 0.04 | P07437 | 4.78 | 49.60 | 9 | Reported as a group | Reported as a group |
| 2 | CCT5 | T-complex protein 1 subunit epsilon | 1.26 | 0.03 | P48643 | 5.4 | 59.50 | 4 | 11.5 | 2.00E-08 |
| 3 | RUVBL-1 | RuvB-like 1 protein | -1.2 | 0.0097 | Q9Y265 | 6.02 | 50.20 | 5 | 19.7 | 1.00E-10 |
| 4 | GAPDH | Glyceraldehyde-3-phosphate dehydrogenase | 1.29 | 0.004 | P04406 | 8.58 | 35.90 | 5 | 15.2 | 1.00E-10 |
| 5 | UQCRFS1 | Cytochrome b-c1 complex subunit Rieske, mitochondrial | -1.2 | 0.0034 | P47985 | 11.3 | 8.00 | 3 | 10.6 | 1.00E-06 |
| 6 | PRDX3 | Thioredoxin-dependent peroxide reductase, mitochondrial | 1.2 | 0.023 | P30048 | 5.77 | 21.40 | 5 | 19.1 | 1.00E-10 |
| 7 | IMPDH2 | Inosine 5' monophosphate dehydrogenase 2 | -1.2 | 0.04 | P12268 | 6.46 | 55.60 | 4 | 9.3 | 1.00E-08 |
| 8 | ANXA2P2 | Putative Annexin A2 | 1.22 | 0.04 | A6NMY6 | 6.48 | 38.50 | 3 | 8.9 | 1.00E-06 |
| 9 | CLIC1 | Chloride intracellular channel protein 1 | 1.22 | 0.013 | O00299 | 5.09 | 26.80 | 3 | 19.9 | 1.00E-06 |
| 10 | UCHL1 | Ubiquitin carboxyl-terminal hydrolase isozyme L1 | -1.2 | 0.025 | P09936 | 5.2 | 24.50 | 5 | 33.2 | 1.00E-10 |
| 11 | COX5A | Cytochrome c oxidase subunit 5A, mitochondrial | -1.2 | 0.04 | P20674 | 4.9 | 12.50 | 2 | 15.3 | 1.00E-04 |

| Sample ID (60µM) | Abbrev | Protein ID | Fold  change | T-test | Accession No | pI | MW(kDa) | Matched peptides | % Coverage | | P value |
| --- | --- | --- | --- | --- | --- | --- | --- | --- | --- | --- | --- |
| 1 | P4HB | Protein disulfide-isomerase; A1 | 1.2 | 0.028 | P07237 | 4.69 | 55.20 | 15 | | 32.1 | 3.00E-30 |
| 2 | HNRNPH1 | Heterogeneous nuclear ribonucleoprotein H | -1.2 | 0.013 | P31943 | 5.89 | 49.10 | 3 | | 12.5 | 1.00E-06 |
| 3 | TPM4 | Tropomyosin alpha-4 chain | -1.2 | 0.018 | P67937 | 4.67 | 28.30 | 6 | | 14.9 | 1.00E-12 |
| 4 | CLIC1 | Chloride intracellular channel protein 1 | -1.2 | 0.04 | O00299 | 5.09 | 26.70 | 4 | | 23.2 | 1.00E-08 |
| 5 | PPA1 | Inorganic pyrophosphatase | -1.2 | 0.027 | Q15181 | 5.5 | 32.60 | 6 | | 26.0 | 4.00E-12 |
| 6 | GSTP1 | Glutathione S-transferase P | -1.27 | 0.02 | P09211 | 5.44 | 23.20 | 6 | | 40.5 | 2.00E-12 |
| 7 | PRDX6 | Peroxiredoxin-6 | 1.2 | 0.001 | P30041 | 6.02 | 24.80 | 4 | | 22.8 | 1.00E-08 |
| 8 | ACADM | Medium chain specific acylCoA dehydrogenase Mito | 1.41 | 0.0029 | P11310 | 7.02 | 43.60 | 3 | | 10.9 | 1.00E-06 |
| 9 | ANXA2P2 | Putative annexin A2-like protein | 1.24 | 0.02 | A6NMY6 | 6.48 | 38.50 | 3 | | 8.9 | 1.00E-06 |
| 10 | ALDOA | Fructose -bisphosphate aldolase A | 1.26 | 0.016 | P04075 | 8.39 | 39.20 | 5 | | 26.9 | 1.00E-10 |
| 11 | PSMA5 | Proteasome subunitl alpha type 5 | -1.27 | 0.02 | P28066 | 4.74 | 26.30 | 4 | | 22.8 | 1.00E-08 |
| 12 | ARHGDIA | Rho GDP-dissociation inhibitor 1 | -1.2 | 0.025 | P52565 | 5.01 | 23.00 | 4 | | 34.3 | 1.00E-08 |
| 13 | PRDX2 | Peroxiredoxin 2 | -1.2 | 0.041 | P32119 | 5.67 | 21.70 | 1 | | 5.6 | 1.00E-02 |
| 14 | HBB | Hemoglobin subunit beta | -1.29 | 0.031 | P68871 | 6.81 | 15.80 | 6 | | 6.8 | 1.00E-10 |

| Sample ID  (75µM) | Abbrev | | Protein ID | Fold change | | T-test | Accession No | | pI | MW(kDa) | | Matched peptides | | % Coverage | P value |  |
| --- | --- | --- | --- | --- | --- | --- | --- | --- | --- | --- | --- | --- | --- | --- | --- | --- |
| 1 | | EF2 | Elongation factor 2 | | -1.22 | 0.04 | P13639 | 6.42 | | | 95.10 | 2 | 2.214 | | 9.00E-04 | |
| 2 | | FUBP2 | Far upstream element-binding protein 2 | | 1.2 | 0.013 | Q92945 | 6.85 | | | 73.10 | 6 | 10.83 | | 8.00E-12 | |
| 3 | | LMNA | Prelamin-A/C | | 1..21 | 0.013 | P02545 | 6.57 | | | 73.70 | 10 | 20.181 | | 2.00E-20 | |
| 4 | | LMNB2 | Lamin-B2 | | -1.2 | 0.0076 | Q03252 | 5.29 | | | 67.10 | 5 | 8.833 | | 1.00E-10 | |
| 5 | | P4HB | Protein disulfide isomerase A1 | | 1.24 | 0.0058 | P07237 | 4.69 | | | 55.30 | 15 | 32.087 | | 3.00E-30 | |
| 6 | | HNRNPK | Heterogeneous nuclear ribonucleoprotein K | | 1.2 | 0.0068 | P61978 | 5.39 | | | 50.90 | 6 | 20.609 | | 1.00E-12 | |
| 7 | | TUBB | Tubulin chain | | -1.33 | 0.0092 | P07437 | 4.78 | | | 49.60 | 9 | Reported as a group | | Reported as a group | |
| 8 | | RUVBL1 | RuvB-like 1 | | -1.4 | 0.00015 | Q9Y265 | 6.02 | | | 15.20 | 5 | 19.737 | | 1.00E-10 | |
| 9 | | PDIA3 | Protein disulfide-isomerase A3 | | 1.2 | 0.007 | P30101 | 5.61 | | | 54.20 | 7 | 16.634 | | 1.00E-06 | |
| 10 | | ENO1 | Alpha-enolase | | 1.5 | 0.0013 | P06733 | 6.99 | | | 47.00 | 4 | 13.594 | | 1.00E-08 | |
| 11 | | ENO2 | Enolase | | 1.4 | 0.0014 | P09104 | 4.9 | | | 47.10 | 5 | 21.483 | | 1.00E-10 | |
| 12 | | ANXA11 | Annexin A11 | | -1.2 | 0.019 | P50995 | 7.53 | | | 54.35 | 5 | 10.297 | | 1.00E-10 | |
| 13 | | RPLPO | 60S acidic ribosomal protein P0 | | -1.3 | 0.009 | P05388 | 5.7 | | | 34.25 | 9 | 34.069 | | 4.00E-18 | |
| 14 | | HNRNPH3 | Heterogeneous nuclear ribonucleoprotein H3 | | -1.26 | 0.009 | P31942 | 6.37 | | | 36.90 | 4 | 17.341 | | 1.00E-08 | |
| 15 | | ANXA2 | Annexin A2 | | 1.22 | 0.027 | P07355 | 7.56 | | | 38.40 | 9 | 28.909 | | 1.00E-18 | |
| 16 | | PPA1 | Inorganic pyrophosphatase | | -1.27 | 0.008 | Q15181 | 5.5 | | | 32.60 | 6 | 25.952 | | 4.00E-12 | |
| 17 | | SRSF1 | Splicing factor, arginine/serine-rich 1 | | -1.3 | 0.002 | Q07955 | 10.37 | | | 27.60 | 3 | 14.286 | | 1.00E-06 | |
| 18 | | CLIC1 | Chloride intracellular channel protein 1 | | 1.26 | 0.002 | O00299 | 5.09 | | | 26.77 | 4 | 23.237 | | 1.00E-08 | |
| 19 | | PRDX6 | Peroxiredoxin-6 | | -1.25 | 0.008 | P30041 | 6.02 | | | 24.88 | 4 | 22.768 | | 1.00E-08 | |
| 20 | | UQCRFS1 | Cytochrome b-c1 complex subunit Rieske, mitochondrial | | -1.4 | 1.7x10^-5^ | P47985 | 11.3 | | | 8.06 | 3 | 10.584 | | 1.00E-06 | |
| 21 | | YWHAZ | 14-3-3 protein zeta/delta | | -1.2 | 0.005 | P63104 | 4.73 | | | 27.70 | 2 | 8.98 | | 1.00E-04 | |
| 22 | | GSTP1 | Glutathione S-transferase P | | 1.21 | 0.0007 | P09211 | 5.44 | | | 23.06 | 6 | 40.476 | | 2.00E-12 | |
| 23 | | PRDX2 | Peroxiredoxin-2 | | -1.24 | 0.008 | P32119 | 5.67 | | | 21.70 | 5 | 25.989 | | 2.00E-10 | |
| 24 | | TBCA | Tubulin-specific chaperone A | | -1.2 | 0.0045 | O75347 | 5.25 | | | 12.70 | 3 | 18.519 | | 1.00E-06 | |
| 25 | | HBB | Hemoglobin subunit beta | | 1.41 | 0.002 | P68871 | 6.81 | | | 15.80 | 6 | 53.793 | | 1.00E-12 | |
| 26 | | COX5A |  | | -1.3 | 0.008 | P20674 | 4.88 | | | 12.49 | 2 | 15.333 | | 1.00E-04 | |
| 27 | | KHSRP | Far upstream element binding protein 2 | | 1.21 | 0.007 | Q92945 | 6.85 | | | 73.00 | 8 | 16.0 | | 1.00E-16 | |
| 28 | | STIP1 | Stress- induced phosphoprotein 1 | | -1.2 | 0.025 | P31948 | 6.4 | | | 62.59 | 9 | 14.9 | | 4.00E-18 | |
| 29 | | KRT8 | Keratin type II cytoskeletal 8 | | 1.2 | 0.028 | P05787 | 5.52 | | | 53.50 | 9 | 27.3 | | 1.00E-18 | |
| 30 | | IMPDH2 | Inosine 5' monophosphate dehydrogenase 2 | | -1.26 | 0.04 | P12268 | 6.46 | | | 55.60 | 4 | 9.3 | | 1.00E-08 | |
| 31 | | PHGDH | D-3-phosphoglycerate dehydrogenase | | -1.25 | 0.012 | O43175 | 6.31 | | | 56.40 | 3 | 5.3 | | 1.00E-06 | |
| 32 | | ANXA11 | Annexin A11 | | -1.2 | 0.019 | P50995 | 7.53 | | | 54.35 | 5 | 10.3 | | 1.00E-10 | |
| 33 | | HNRNPH1 | Heterogeneous ribonucleoprotein H | | -1.2 | 0.04 | P31943 | 5.89 | | | 49.10 | 3 | 12.0 | | 1.00E-06 | |
| 34 | | ACADM | Medium chain specific acylCoA dehydrogenase Mito | | 1.5 | 5.3x10^-5^ | P11310 | 7.02 | | | 43.60 | 3 | 10.9 | | 1.00E-06 | |
| 35 | | ALDOA | Fructose bisphosphate aldolase A | | 1.2 | 0.028 | P04075 | 8.39 | | | 39.20 | 5 | 26.9 | | 1.00E-10 | |
| 36 | | STOML2 | Stomatin like protein 2 | | -1.25 | 0.0001 | Q9UJZ1 | 6.87 | | | 38.50 | 3 | 13.5 | | 2.00E-06 | |
| 37 | | TPT1 | Translationally controlled tumor protein | | 1.2 | 0.0015 | P13693 | 4.84 | | | 19.58 | 3 | 22.7 | | 1.00E-06 | |

| Sample ID (100µM) | Abbrev | Protein ID | Fold change | T-test | Accession No | pI | MW(kDa) | Matched peptides | % Coverage | P value |
| --- | --- | --- | --- | --- | --- | --- | --- | --- | --- | --- |
| 1 | EF2 | Elongation factor 2 | -1.26 | 0.02 | P13639 | 6.42 | 95.10 | 2 | 2.2 | 9.0E-04 |
| 2 | KHSRP | Far upstream element-binding protein 2 | 1.24 | 0.0039 | Q92945 | 6.85 | 73.00 | 6 | 10.8 | 8.0E-12 |
| 3 | LMNA | Prelamin-A/Cprecursor | 1.28 | 0.0024 | P02545 | 6.57 | 71.70 | 10 | 20.2 | 4.0E-20 |
| 4 | LMNB2 | Lamin-B2 | -1.2 | 0.04 | Q03252 | 5.29 | 67.10 | 5 | 8.8 | 1.0E-10 |
| 5 | PURG | Bifunctional purine biosynthesis protein | -1.2 | 0.03 | P31939 | 6.27 | 64.50 | 3 | 6.6 | 4.0E-06 |
| 6 | PDIA3 | Protein disulfide-isomerase A3 | -1.22 | 0.006 | P30101 | 5.61 | 54.20 | 8 | 33.47 | 3.00E-24 |
| 7 | HNRNPK | Heterogeneous nuclear ribonucleoprotein K | 1.4 | 0.003 | P61978 | 5.39 | 50.90 | 6 | 20.6 | 1.0E-12 |
| 8 | HNRNPL | Heterogeneous nuclear ribonucleoprotein L | 1.2 | 0.009 | P14866 | 8.46 | 64.00 | 11 | 27.8 | 4.0E-22 |
| 9 | VIM | Vimentin | 1.3 | 0.033 | P08670 | 5.05 | 53.48 | 10 | 27.7 | 3.0E-20 |
| 10 | P4HB | Protein disulfide isomerase A1 | 1.2 | 0.005 | P07237 | 4.69 | 55.25 | 15 | 32.1 | 3.0E-30 |
| 11 | TUBB | Tubulin chain | -1.5 | 0.0024 | P07437 | 4.78 | 49.60 | 9 | Reported  as Group | Reported as Group |
| 12 | IMPDH2 | Inosine-5'-monophosphate dehydrogenase 2 | -1.4 | 0.005 | P12268 | 6.46 | 5.60 | 3 | 5.6 | 1.0E-06 |
| 13 | VIM | Vimentin | 1.2 | 0.004 | P08670 | 5.05 | 53.40 | 4 | 11.2 | 1.0E-08 |
| 14 | FSCN1 | Fascin | -1.24 | 0.009 | Q16658 | 6.81 | 54.30 | 3 | 6.9 | 1.0E-06 |
| 15 | RUVBL1 | RuvB-like 1;cytosolic protein | -1.6 | 0.009 | Q9Y265 | 6.02 | 50.19 | 5 | 19.7 | 1.0E-10 |
| 16 | PDIA3 | Protein disulfide-isomerase A3 | 1.3 | 0.0092 | P30101 | 5.61 | 54.20 | 7 | 16.6 | 2.0E-14 |
| 17 | HNRNPH | Heterogeneous nuclear ribonucleoprotein H | 1.2 | 0.009 | P31943 | 5.89 | 49.10 | 8 | 14.3 | 1.0E-16 |
| 18 | ENO2 | Enolase | 1.4 | 0.0006 | P09104 | 4.9 | 47.10 | 5 | 21.5 | 1.0E-10 |
| 19 | ENO1 | Alpha-enolase | -1.3 | 0.012 | P06733 | 6.99 | 47.00 | 12 | 40.3 | 2.0E-24 |
| 20 | FLOT1 | Flotillin-1 | -1.3 | 0.005 | O75955 | 7.08 | 47.35 | 4 | 13.3 | 1.0E-08 |
| 21 | TUFM | Elongation factor Tu, mitochondrial | -1.2 | 0.029 | P49411 | 6.3 | 45.00 | 13 | 34.7 | 1.0E-26 |
| 22 | 2-Sep | Septin-2 | 1.25 | 0.009 | Q15019 | 6.15 | 41.46 | 8 | 29.6 | 2.0E-16 |
| 23 | CAPG | Macrophage-capping protein | 1.2 | 0.006 | P40121 | 5.8 | 38.40 | 1 | 3.9 | 1.0E-02 |
| 24 | HNRNPH3 | Heterogeneous nuclear ribonucleoprotein H3 | -1.3 | 0.003 | P31942 | 6.37 | 38.40 | 4 | 17.3 | 1.0E-08 |
| 25 | RPLPO | 60S acidic ribosomal protein P0 | -1.5 | 0.008 | P05388 | 5.7 | 34.20 | 9 | 34.1 | 4.0E-18 |
| 26 | ANXA2 | Annexin A2 | 1.32 | 0.002 | P07355 | 7.56 | 38.40 | 16 | 45.4 | 1.0E-32 |
| 27 | PPA1 | Inorganic pyrophosphatase | -1.6 | 0.007 | Q15181 | 5.5 | 38.40 | 6 | 26.0 | 4.0E-12 |
| 28 | TPM3 | Tropomyosin alpha-3 chain | -1.3 | 0.002 | P06753 | 4.68 | 38.40 | 7 | 22.6 | 1.0E-14 |
| 29 | SRSF1 | Splicing factor, arginine/serine-rich 1 | -1.5 | 0.0006 | Q07955 | 10.37 | 32.60 | 3 | 29.6 | 1.0E-06 |
| 30 | TPM4 | Tropomyosin alpha-4 chain | -1.4 | 0.0081 | P67937 | 4.67 | 28.30 | 9 | 25.4 | 1.0E-18 |
| 31 | CLIC1 | Chloride intracellular channel protein 1 | 1.3 | 0.0001 | O00299 | 5.09 | 26.70 | 4 | 23.2 | 1.0E-08 |
| 32 | PHB | Prohibitin | 1.4 | 0.0015 | P35232 | 5.57 | 28.30 | 11 | 44.9 | 1.0E-22 |
| 33 | PGAM4 | Phosphoglycerate mutase 4 | -1.3 | 0.002 | Q8N0Y7 | 6.19 | 26.70 | 6 | 29.1 | 1.0E-12 |
| 34 | HNRNPHC | Heterogeneous nuclear ribonucleoproteins C1/C2 | -1.3 | 0.004 | P07910 | 5.0 | 29.70 | 2 | 9.1 | 1.0E-04 |
| 35 | PRDX6 | Peroxiredoxin-6 | -1.6 | 0.0001 | P30041 | 6.02 | 28.70 | 4 | 22.8 | 1.0E-08 |
| 36 | YWHAZ | 14-3-3 protein zeta/delta | -1.4 | 0.0002 | P63104 | 4.73 | 24.80 | 2 | 9.0 | 1.0E-04 |
| 37 | TPI1 | Triosephosphate Isomerase | -1.21 | 0.0025 | P60174 | 5.65 | 30.70 | 6 | 34.27 | 1.00E-14 |
| 38 | HSD17B10 | 3-hydroxyacyl-CoA dehydrogenase type-2 | -1.25 | 0.002 | Q99714 | 7.87 | 27.70 | 6 | 28.7 | 1.0E-12 |
| 39 | PSMB3 | Proteasome subunit beta type-3 | -1.3 | 0.0012 | P49720 | 6.12 | 24.20 | 2 | 16.6 | 1.0E-04 |
| 40 | PRDX2 | Peroxiredoxin-2 | -1.5 | 0.0005 | P32119 | 5.67 | 34.90 | 5 | 26.0 | 2.0E-10 |
| 41 | NME2 | Nucleoside diphosphate kinase B | -1.4 | 0.018 | P22392 | 8.55 | 17.15 | 2 | 14.5 | 1.0E-04 |
| 42 | TBCA | Tubulin-specific chaperone A | -1.3 | 0.04 | O75347 | 5.25 | 12.70 | 3 | 18.5 | 1.0E-06 |
| 43 | HBB | Hemoglobin subunit beta | 1.5 | 0.0028 | P68871 | 6.81 | 21.70 | 6 | 53.8 | 1.0E-12 |
| 44 | COX5A | Cytochrome c oxidase subunit 5A, mitochondrial | -1.3 | 0.029 | P20674 | 4.88 | 17.15 | 2 | 15.3 | 1.0E-04 |
| 45 | KHSRP | Far upstream element binding protein 2 | -1.2 | 0.025 | Q92945 | 6.85 | 73.00 | 8 | 16.0 | 1.00E-16 |
| 46 | TRAP1 | Heat shock protein 75 kda mitochondrial | 1.2 | 0.04 | Q12931 | 6.1 | 73.50 | 9 | 16.6 | 1.00E-18 |
| 47 | FUBP1 | Far upstream element binding protein 1 | -1.26 | 0.04 | Q96AE4 | 7.33 | 67.38 | 7 | 13.2 | 1.00E-14 |
| 48 | STIP1 | Stress- induced phosphoprotein 1 | -1.32 | 0.002 | P31948 | 6.4 | 62.59 | 8 | 14.9 | 1.00E-16 |
| 49 | PDIA3 | Protein disulfide isomerase A3 | 1.2 | 0.0003 | P30101 | 5.61 | 54.20 | 12 | 33.5 | 3.00E-24 |
| 50 | KRT8 | Keratin type II cytoskeletal 8 | 1.2 | 0.007 | P05787 | 5.52 | 53.50 | 9 | 27.3 | 1.00E-18 |
| 51 | IMPDH2 | Inosine 5' monophosphate dehydrogenase 2 | 1.3 | 0.004 | P12268 | 6.46 | 55.60 | 4 | 9.3 | 1.00E-08 |
| 52 | PHGDH | D-3-phosphoglycerate dehydrogenase | -1.4 | 0.002 | O43175 | 6.31 | 56.40 | 3 | 5.3 | 1.00E-06 |
| 53 | HNRNPH1 | Heterogeneous ribonucleoprotein H | -1.2 | 0.0023 | P31943 | 5.89 | 49.19 | 3 | 12.0 | 1.00E-06 |
| 54 | KRT7 | Keratin type II cytoskeletal 7 | 1.5 | 0.023 | P08729 | 5.39 | 51.20 | 16 | 39.7 | 1.00E-32 |
| 55 | KRT18 | Keratin type 1 cytoskeletal 18 | -1.2 | 0.04 | P05783 | 5.34 | 47.90 | 9 | 25.6 | 1.00E-18 |
| 56 | ACTG1 | Actin cytoplasmic (protein group) | -1.21 | 0.03 | P63261 | 5.31 | 41.70 | 7 | 25.9 | 1.00E-14 |
| 57 | ACADM | Medium chain specific acylCoA dehydrogenase Mito | 1.65 | 0.002 | P11310 | 7.02 | 43.60 | 3 | 10.9 | 1.00E-06 |
| 58 | SEPT2. | Septin-2 | -1.2 | 0.008 | Q15019 | 6.15 | 41.50 | 4 | 14.4 | 1.00E-08 |
| 59 | ALDOA | Fructose bisphosphate aldolase A | 1.27 | 0.011 | P04075 | 8.39 | 39.20 | 5 | 26.9 | 1.00E-10 |
| 60 | STOML2 | Stomatin like protein 2 | -1.5 | 0.0013 | Q9UJZ1 | 6.87 | 38.50 | 3 | 13.5 | 2.00E-06 |
| 61 | DNAJB11 | DNA J homolog subfamily B member 11 | -1.3 | 0.06 | Q9UBS4 | 5.81 | 38.14 | 5 | 6.4 | 1.00E-10 |
| 62 | ANXA11 | Annexin A1 | 1.2 | 0.015 | P04083 | 6.64 | 38.60 | 12 | 40.8 | 1.00E-24 |
| 63 | CAP2B | F-actin capping protein subunit beta | -1.28 | 0.008 | P47756 | 5.36 | 31.20 | 3 | 8.7 | 1.00E-04 |
| 64 | ARHGDIA | Rho GDP dissociation inhibitor 1 | -1.5 | 0.004 | P52565 | 5.01 | 23.10 | 4 | 34.3 | 1.00E-04 |
| 65 | GSTP1 | Glutathione-S transferase | 1.3 | 0.00021 | P09211 | 5.44 | 23.20 | 6 | 40.5 | 2.00E-12 |
| 66 | KRT2 | Keratin type II cytoskeletal 2 epidermal | 1.2 | 0.007 | P35908 | 8.07 | 12.72 | 2 | 4.1 | 1.00E-04 |

**Table B: Comparisons of the top diseases and disorders, molecular and cell functions, canonical pathways and toxlists identified using IPA analysis of differentially expressed proteins identified using 2-DE protein profiling approach from BEAS-2B cells treated with 30, 60, 75 and 100 µM of Ni (II).** Only the top two identifiers for each categories were listed in the Table.

|  | 30 µM | 60 µM | 75 µM | 100 µM |
| --- | --- | --- | --- | --- |
| Top Diseases and Disorders | Cancer | Cancer | Cancer | Cancer |
|  | Dermatological diseases and conditions | Dermatological diseases and conditions | Gastrointestinal Disease | Gastrointestinal disease |
| Top Molecular and Cell functions | Cell death | Small molecule biochemistry | Cell death | Cell death |
|  | Small molecule biochemistry | Cell death | Small molecule biochemistry | Small molecule biochemistry |
| Top Canonical Pathways | Mitochondrial dysfunction | Phenylalanine metabolism | Glycolysis/ gluconeogenesis | Glycolysis/ gluconeogenesis |
|  | Pentose and glucuronate interconversions | Glutathione metabolism | 14-3-3 mediated signaling | 14-3-3 mediated signaling |
| Top Toxlists | Mitochondrial dysfunction | Oxidative stress | Oxidative stress | Oxidative stress |
|  | Oxidative stress | Hypoxia inducible factor signaling | Mitochondrial dysfunction | NRF2-mediated oxidative stress response |
